# Experimental assessment of pelvis slipping during postless traction for orthopaedic applications

**DOI:** 10.1186/s13018-024-04704-0

**Published:** 2024-04-01

**Authors:** Marco Daghero, Simone Borrelli, Taian M. Vieira, Francesco Cannito, Alessandro Aprato, Andrea Audisio, Cristina Bignardi, Mara Terzini

**Affiliations:** 1 Department of Mechanical and Aerospace Engineering, Politecnico di Torino, Turin, Italy; 2 LISiN-Department of Electronics and Telecommunications, Politecnico di Torino, Turin, Italy; 3 PolitoBIOMed Lab, Politecnico di Torino, Turin, Italy; 4 Department of Surgical Sciences, University of Turin, Turin, Italy

**Keywords:** Hip arthroscopy, Femur fracture, Postless technique, Postless traction, High-friction pads, Trendelenburg position, PinkPad, CarePad

## Abstract

**Background:**

The application of lower limb traction during hip arthroscopy and femur fractures osteosynthesis is commonplace in orthopaedic surgeries. Traditional methods utilize a perineal post on a traction table, leading to soft tissue damage and nerve neuropraxia. A postless technique, using high-friction pads, has been considered as a potential damage-free alternative. However, whether these pads sufficiently prevent patient displacement remains unknown. Thus, this study systematically assesses the efficacy of commercial high-friction pads (PinkPad and CarePad) in restraining subject displacement, for progressively increasing traction loads and different Trendelenburg angles.

**Methods:**

Three healthy male subjects were recruited and tested in supine and Trendelenburg positions (5° and 10°), using a customized boot-pulley system. Ten load disks (5 kg) were dropped at 15s intervals, increasing gradually the traction load up to 50 kg. Pelvis displacement along the traction direction was measured with a motion capture system. The displacement at 50 kg of traction load was analyzed and compared across various pads and bed inclinations. Response to varying traction loads was statistically assessed with a quadratic function model.

**Results:**

Pelvis displacement at 50 kg traction load was below 60 mm for all conditions. Comparing PinkPad and CarePad, no significant differences in displacement were observed. Finally, similar displacements were observed for the supine and Trendelenburg positions.

**Conclusions:**

Both PinkPad and CarePad exhibited nearly linear behavior under increasing traction loads, limiting displacement to 60 mm at most for 50 kg loads. Contrary to expectations, placing subjects in the Trendelenburg position did not increase adhesion.

**Supplementary Information:**

The online version contains supplementary material available at 10.1186/s13018-024-04704-0.

## Background

In the context of orthopaedic surgical interventions, procedures such as hip arthroscopy or femur fractures’ osteosynthesis are common and in high demand [1, 2]. In both cases the application of lower limb traction is required. During hip arthroscopy the application of a traction load permits to achieve sufficient distraction of the hip joint [3]. Similarly, traction is a well-established technique to achieve indirect reduction of bone fragments in femur fractures [4]. Lower limb traction is usually performed with a standard traction table equipped with a perineal post which prevents the patient’s slipping, and thus, guarantees the necessary counter-traction.

Nevertheless, several complications emerge related to the high perineal pressure resulting during the hip distraction [5–7]. Main reported complications include soft tissue damage, pudendal nerve neuropraxia and sciatic nerve neuropraxia [8], which may lead to urinary and fecal continence disorders, chronic pain, and sexual function disorders [9]. To contend with these issues, a novel commercial high-friction conformable pad (PinkPad; Xodus Medical Inc., USA) was introduced as alternative to the perineal post. Mei-Dan et al. [10] reported for the first time a postless distraction during hip arthroscopy surgery. Promising results in terms of safety and efficacy were then confirmed by subsequent studies [10–12]. More recently, the postless distraction was introduced as a reduction tool for femur fractures’ nailing [13, 14]. However, these works reported functional evaluations in a qualitative manner and the displacement of the patient during surgery was not monitored. Indeed, a limited patient’s slipping is essential for achieving a successful distraction of the hip joint or reduction of fracture’s fragments.

To the best of the authors’ knowledge, the available literature lacks any assessment of the pad performance in terms of patient’s displacement within a controlled traction environment. Therefore, the aim of this study is to measure the displacement of subjects lying on high-friction pads while subjected to an increasing traction load at different Trendelenburg angles. Then, two commercial pads are compared: the PinkPad, currently considered the standard in postless orthopaedic interventions, and the CarePad (Ab Medica S.P.A., Italy). This evaluation was conducted to characterize and compare both pads’ effectiveness in preventing patient’s slipping, as a first step towards their future standardization in orthopaedic surgeries that require traction.

## Methods

### Experimental protocol

Three healthy male subjects were recruited to participate in this study after providing written informed consent according to the Declaration of Helsinki. The subjects had comparable baseline characteristics (Table 1) to minimize potential confounding variables, facilitating the examination of the specific factors under investigation and strengthening the overall robustness of the experimental design. The experimental protocol was approved by the local Ethical Committee (n°: 842.924).

**Table 1:** Body mass, height and body mass index (BMI) of the enlisted subjects

ID subject	Body mass (kg)	Height (cm)	BMI (kg/m^2^)
S1	73	177	23.3
S2	73	182	22.0
S3	73	178	23.0

**Fig. 1:**
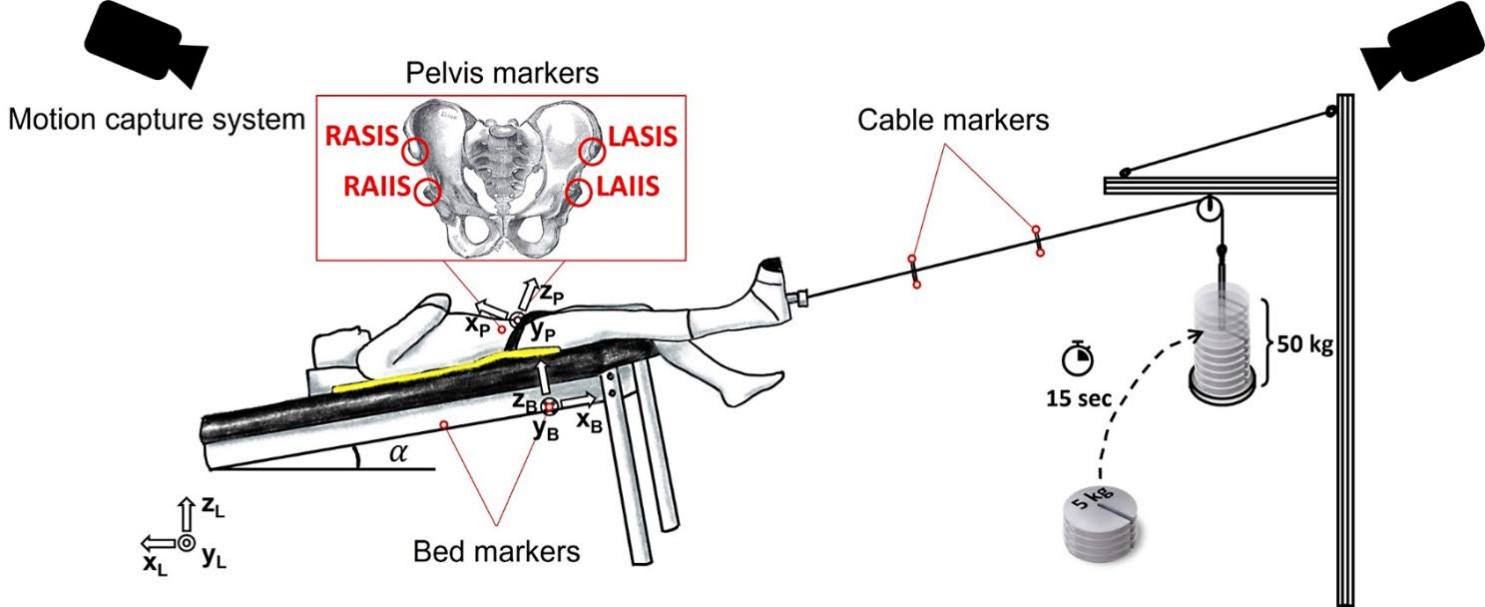
Representation of the experimental setup

Each subject was tested on a padded bed, in supine and in two Trendelenburg positions (α: 5° and 10°). The test procedure consisted in applying traction of progressively greater magnitude coaxially to the longitudinal axis of the subject’s right leg through a customized, boot-pulley system (Fig. 1). The system included a traction boot connected via a steel cable to a pulley allowing for precise control of the traction load by attaching a set of load disks (5 kg). Ten load disks were added at 15s intervals, each at a time, increasing gradually the traction load up to 50 kg. Subjects’ three-dimensional motion was captured by 12 infrared cameras (Vero v2.2, Vicon system, Oxford, UK). Four reflective markers were used to define the bed plane, as reference to assess the subjects’ motion. Moreover, two pairs of markers were placed on the steel cable to monitor whether the direction of traction was aligned with the bed longitudinal axis. Four markers were placed over the subject’s pelvis, at specific anatomical landmarks, adapting an existing protocol [15]. Due to the supine positioning of the subject, all pelvis’ markers were placed over the anterior side (Fig. 1): the two anterior superior iliac spines (ASIS) and the two anterior inferior iliac spines (AIIS). The subject’s movement was quantified as the displacement of the pelvis center of mass (COM). In order to reconstruct the pelvis COM position, a static capture with the subject standing was added during which two additional markers were placed over the left and right posterior superior iliac spines (PSIS). The pelvis COM was defined as the geometric center of the triangle formed by the two ASIS and the midpoint between the two PSIS [16]. For each subject, the test procedure was repeated 6 times: three bed inclinations (0°, 5° and 10°) for both the commercial pads (i.e., PinkPad and CarePad).

### Data analysis

The coordinates of each marker were sampled at 100 Hz and were low-pass filtered with a 4th order, zero-lag Butterworth filter (cut-off frequency at 2 Hz) to reduce noise and artefacts. For each subject, the pelvis reference frame was defined using the anterior markers only, as follows (Fig. 1):

- The origin corresponds to the left ASIS.
- The y_P_ axis is oriented as the line passing through the left ASIS and the right ASIS, with its positive direction from left to right.
- The z_p_ axis lies in the quasi-frontal plane defined by the cross product between the line connecting the left ASIS with the left AIIS and the y axis, with its positive direction forwards.
- The x_p_ axis is orthogonal to the yz plane and its positive direction is proximal.

**Fig. 2:**
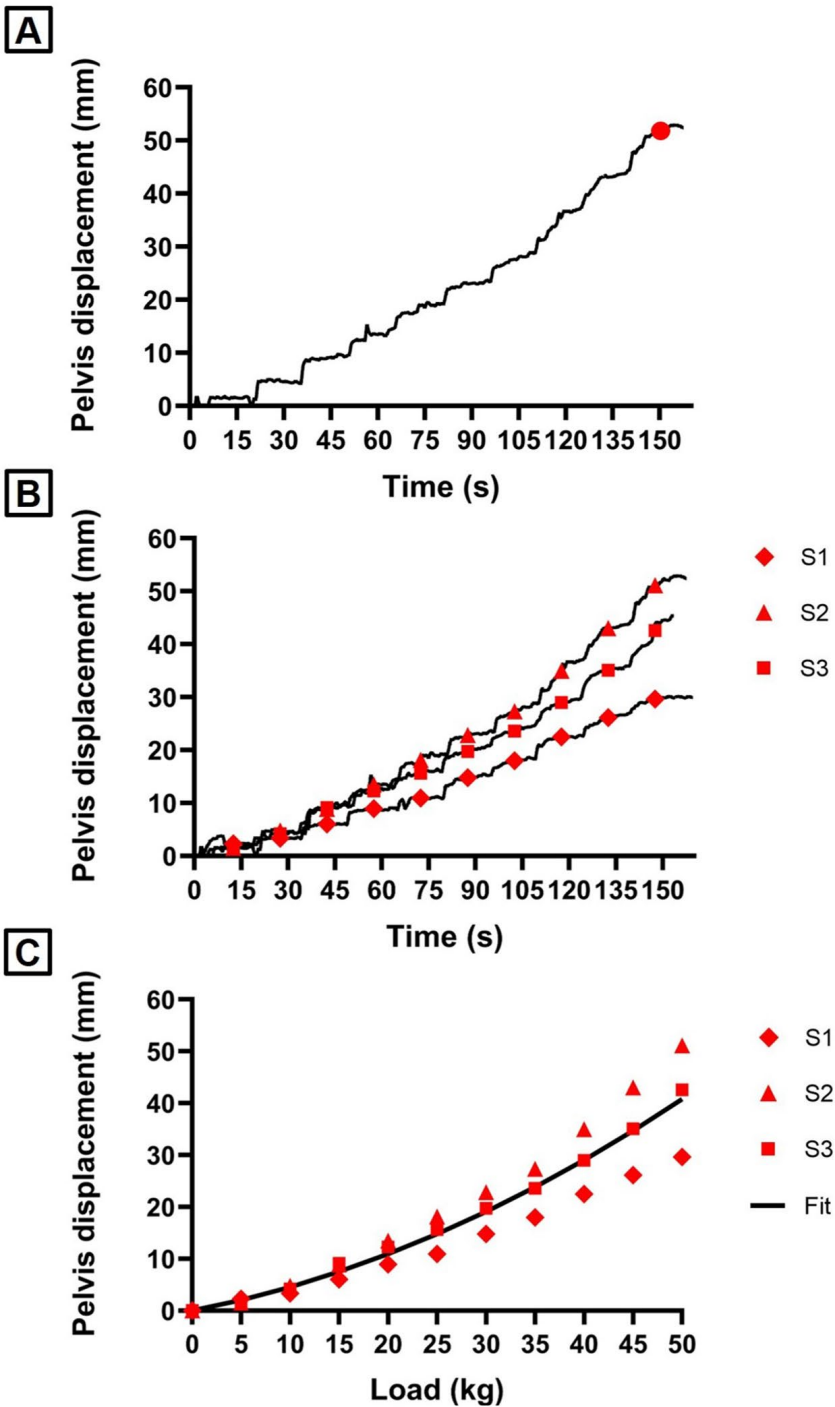
Key steps of the data processing. (**A**): Pelvis displacement along x_B_ axis with the instant corresponding to 50 kg load indicated with a red dot. (**B**): The displacement points identified for each load, overlayed on the pelvis displacement signals of each subject. (**C**): Quadratic regression model of load-displacement points

Then, the position of the pelvis COM with respect to the pelvis reference frame was found from the static capture. Considering the pelvis as a rigid structure, it was assumed that its relative position remained constant. Therefore, it was possible to compute its position during the dynamic tests. The slipping was calculated as the displacement of the pelvis COM along the traction direction, x_B_ (Fig. 1). Figure 2A reports an example of pelvis displacement, with the instant corresponding to the addition of the last 5 kg (50 kg total load) indicated with a red dot. The displacement caused by a total load of 50 kg was analyzed and compared across various pads and bed inclinations. The average displacement and its standard deviation (SD) were evaluated across the three subjects. To characterize and predict the behavior of the pads in terms of the resulting pelvis displacement at different traction loads, a quadratic function was regressed:

$$y=a{x}^{2}+bx+c$$

where $y$ is the pelvis displacement and $x$ is the applied traction load.

The first step consisted in identifying the displacement related to each load from the pelvis displacement signal: this was done dividing the signal in intervals of 5 s before each load was added (starting from the 10–15 s interval) and considering the mean value of each interval (Fig. 2B). For each inclination, the load-displacement points of all the three subjects were used to obtain the fitting curve (Fig. 2C). The first and second order coefficients of the quadratic regression obtained with the different pads and bed inclinations were compared in terms of estimated value and 95% confidence interval (CI).

## Results

**Fig. 3:**
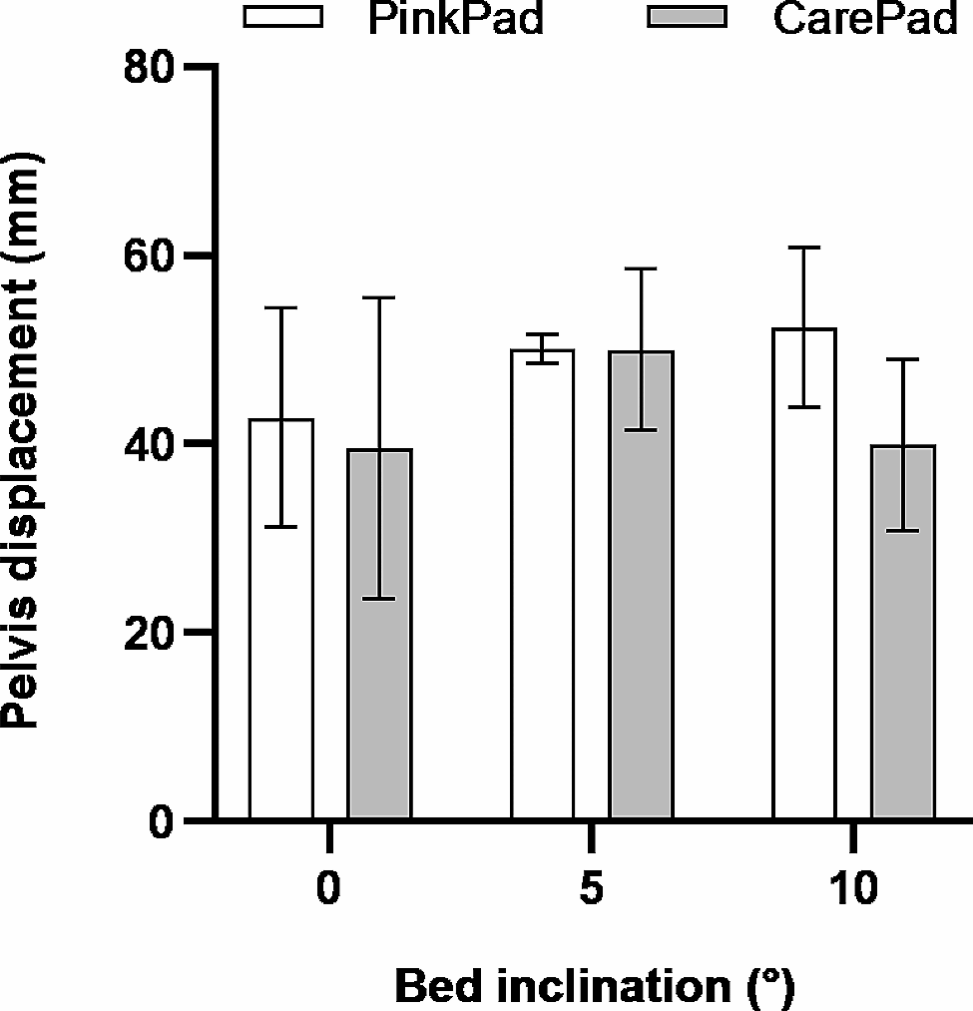
Pelvis displacement of subjects undergoing 50 kg of traction load lying on two different pads with three inclinations of the operating bed. Mean ± SD values of the three subjects are represented

In this study, a total of 18 tests were conducted and in none of them the traction of the lower limb caused a fall of the subject from the bed. Moreover, the angle between the steel cable and the bed longitudinal axis at 50 kg load was 2.1 ± 1.1°, proving the alignment of the traction direction. Figure 3 reports the pelvis displacement at 50 kg of traction load for the two commercial pads at the three bed inclinations. All pads were able to limit pelvis displacement to values lower than 60 mm. By comparing the two pads, no significant difference emerged between the pelvis displacements. Surprisingly, no clear correspondence between pelvis displacement and bed inclination was observed. While a decrease in pelvis displacement might have been expected with greater angles of bed inclination, the Pearson’s correlation coefficient between the two variables (*r* = 0.205) suggested a weak positive correlation, contradicting this initial assumption.

**Fig. 4:**
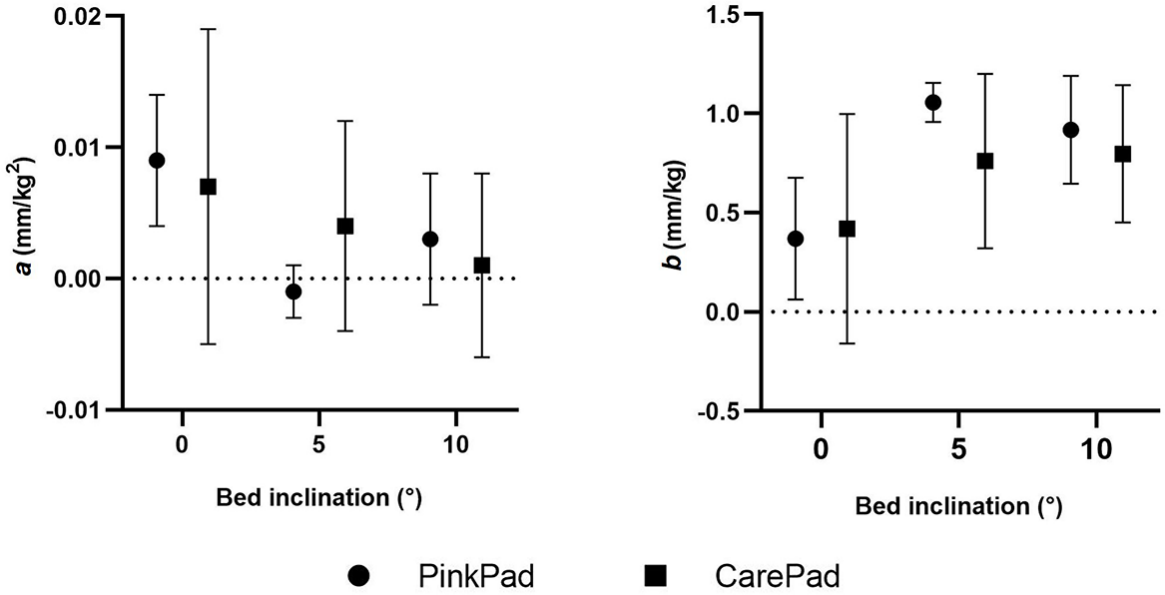
Second (*a*) and first (*b*) order coefficients of the quadratic regression model of load – displacement points at three inclinations of the operating bed. Estimated value and 95% CI of the parameters for the three subjects are represented

Figure 4 presents the second (*a*) and first (*b*) order coefficients of the quadratic regression model of load-displacement points for both pads at different bed inclinations (experimental data, the fitted curves and corresponding equations are reported in Figure S1 of Supplementary material). Similarly to the analysis of the displacement at 50 kg, no significant difference emerged between the coefficients calculated for the different pads. No clear correspondence between the coefficients value and bed inclination was observed. The confidence intervals of the second-order coefficient intersect the zero line under all testing conditions, with the sole exception of the PinkPad at 0°. This suggests a near-linear behavior of the pads when subjected to varying traction loads. Furthermore, the case of the PinkPad at 0° was the only testing condition in which the confidence interval of the zero-order coefficient (intercept) did not intersect the zero line (Figure S2 of Supplementary material).

**Fig. 5:**
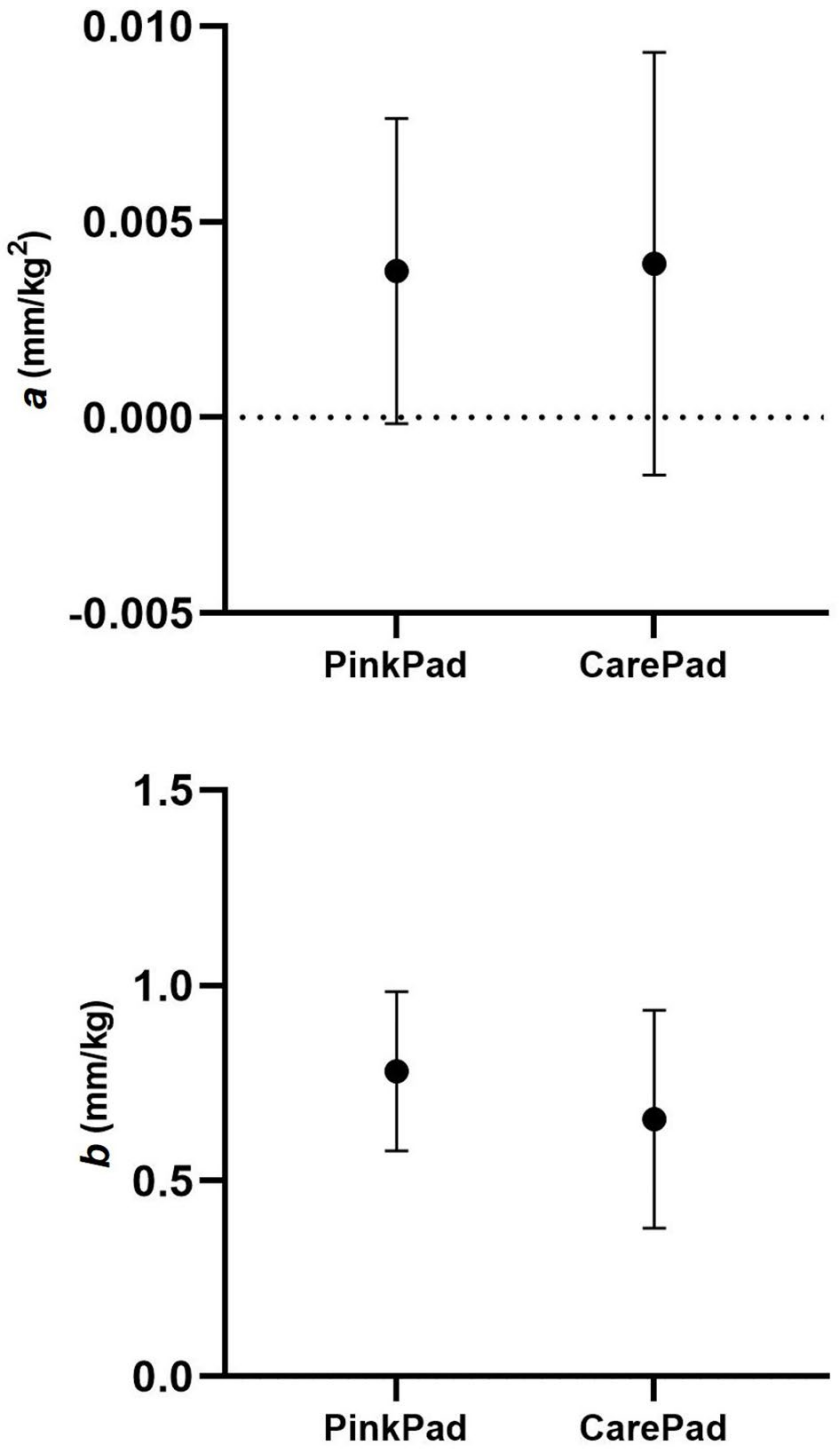
Second (*a*) and first (*b*) order coefficients of the quadratic regression model of load – displacement points considering all inclinations of the operating bed. Estimated value and 95% CI of the parameters for the nine tests are represented

Since the bed inclination did not appear to significantly influence the displacement of the subjects and thus the coefficients obtained from the regressions, a new model was calculated for both the PinkPad and CarePad. This model was obtained following the same methods as the previous regression, but considering all the displacement data points obtained at different bed inclinations. The second (*a*) and first (*b*) order coefficients of these new quadratic regression models are depicted in Fig. 5, suggesting again no significant difference between the two pads (experimental data, fitted curves and equations in Figure S3 of Supplementary material). No complications were reported by the subjects involved in the study.

## Discussion

This novel experimental study revealed a surprising lack of correlation between pelvis displacement and bed inclination. Indeed, papers reporting the adoption of the postless technique make use of an operating bed placed in Trendelenburg positions: at 15–20° in Mei-Dan et al. [10], at 15° in Kollmorgen et al. [11] and at 5–10° in Aprato et al. [14], depending on the patient’s sex, height, weight and fracture type. The choice was motivated by the authors with the aim of increasing gravity and friction and then creating enough resistance between the patient’s upper body and the bed to allow for successful hip distraction [10]. However, when increasing the bed inclination angle, although the component of the weight aligned with the traction direction increases, the resulting normal force decreases and so is the frictional force. The balance between these two components could give a possible explanation to the results obtained in the present study. If further research with a larger group of individuals having varied body compositions confirms this finding, then using the Trendelenburg position in clinical practice may not be necessary.

The threshold for the maximum traction load applied to the subject’s lower limb was set to 50 kg in accordance with prior studies that evaluated the load required for achieving hip distraction or femoral shaft fracture reduction. Regarding hip arthroscopy, the most recent works reported average traction loads between 45 and 50 kg [17–19], while even lower loads, ranging from 30 to 40 kg, proved to be sufficient for femoral shaft fracture reduction [20–22]. More detailed information on traction loads from the literature is available in the Supplementary material (Tables S1 and S2).

By comparing the pelvis displacement at 50 kg of traction load for the two commercial pads, the only difference observed was a slightly higher variability for the CarePad with respect to the PinkPad, as indicated by the average of the standard deviations (27% of the mean vs. 15% of the mean). The same consideration emerged from the quadratic function model, with wider confidence intervals of coefficients *a* (0.011 mm/kg^2^ vs. 0.008 mm/kg^2^) and *b* (0.56 mm/kg vs. 0.41 mm/kg), for the CarePad with respect to the PinkPad.

It should be noted that the current intended use of the CarePad doesn’t specifically include hip arthroscopy or femoral shaft fracture reduction, though it is designed for other surgeries requiring Trendelenburg positioning. However, since its working principle is the same of the PinkPad (both pads aim to prevent patient’s slipping from the operating bed through a high-friction interface), it was chosen to be tested for orthopaedic applications. Finally, both high-friction pads highlighted optimal performance in preventing excessive patient displacement during the application of increasing traction load to its lower limb, with comparable results.

One significant limitation of our study is the homogeneous BMI of our subjects. While selecting subjects with similar BMIs helped to reduce variability in our study, it also poses a limitation. Individuals with higher BMIs were reported to respond differently to postless traction, requiring higher initial traction load [23]. Future studies may investigate how demographic and anatomic factors may affect pelvis slipping and orientation.

Another limitation pertains to the methodology used for assessing pelvis positioning, which was extrapolated based on the spatial position of reflective markers placed on the skin of the subjects. These markers, while practical, only serve as indirect references for underlying bony prominences such as the ASIS and the AIIS. Even though this system was validated in-vitro [24], this indirect measurement might not accurately represent the movement and positioning of the pelvic bone during postless traction. Additionally, the skin, to which these markers are attached, may not move or respond in the same way as the pelvic bone, potentially leading to inaccuracies in our measurements.

Notwithstanding these limitations, this study firstly quantitively assesses the efficacy of high-friction pads, supporting their use in orthopaedic practice and paving the way to the replacement of the perineal post and its related severe complications.

## Conclusions

Ensuring patient safety during postless procedures is crucial in orthopedic surgery. This study quantitatively assessed comparable efficacy of two commercially available high-friction pads (PinkPad and CarePad) in preventing patients’ displacement during the application of increasing traction load to lower limbs.

A quadratic regression model suggested that both pads exhibited nearly linear behaviour when subjected to varying traction loads. This insight may be valuable for clinicians when considering traction loads and how they might relate to patient movement.

Contrary to prevailing beliefs, a lack of strong correlation between bed inclination and pelvis displacement was also recorded. This may imply that in clinical practice, the need to position the bed in a Trendelenburg position could be reconsidered, especially if further research confirms this observation.

### Electronic supplementary material

Below is the link to the electronic supplementary material.

Supplementary Material 1

## Data Availability

The datasets analysed during the current study are available from the corresponding author on reasonable request.

## Abbreviations

ASIS: Anterior superior iliac spine
AIIS: Anterior inferior iliac spine
COM: Center of mass
PSIS: Posterior superior iliac spine
SD: Standard deviation
CI: Confidence interval
BMI: Body mass index
