# Supplementary material for: Experimental assessment of pelvis slipping during postless traction for orthopaedic applications
Source: J Orthop Surg Res. 2024 Apr 1;19:213. doi: 10.1186/s13018-024-04704-0 (PMC10983627; doi:10.1186/s13018-024-04704-0)
Supplement: Supplementary file 1 — Supplementary Material 1 [file 13018_2024_4704_MOESM1_ESM.pdf]

Experimental assessment of pelvis slipping during postless traction for orthopaedic applications

Marco Daghero (1,3), Simone Borrelli (1,3), Taian M. Vieira (2,3), Francesco Cannito (1,3), Alessandro Aprato (4), Andrea Audisio (4), Cristina Bignardi (1,3), Mara Terzini (1,3)

1. Department of Mechanical and Aerospace Engineering, Politecnico di Torino, Turin, Italy.
2. LISiN-Department of Electronics and Telecommunications, Politecnico di Torino, Turin, Italy.
3. Polito[BIO]Med Lab, Politecnico di Torino, Turin, Italy.
4. Department of Surgical Sciences, University of Turin, Turin, Italy.

Supplementary Material

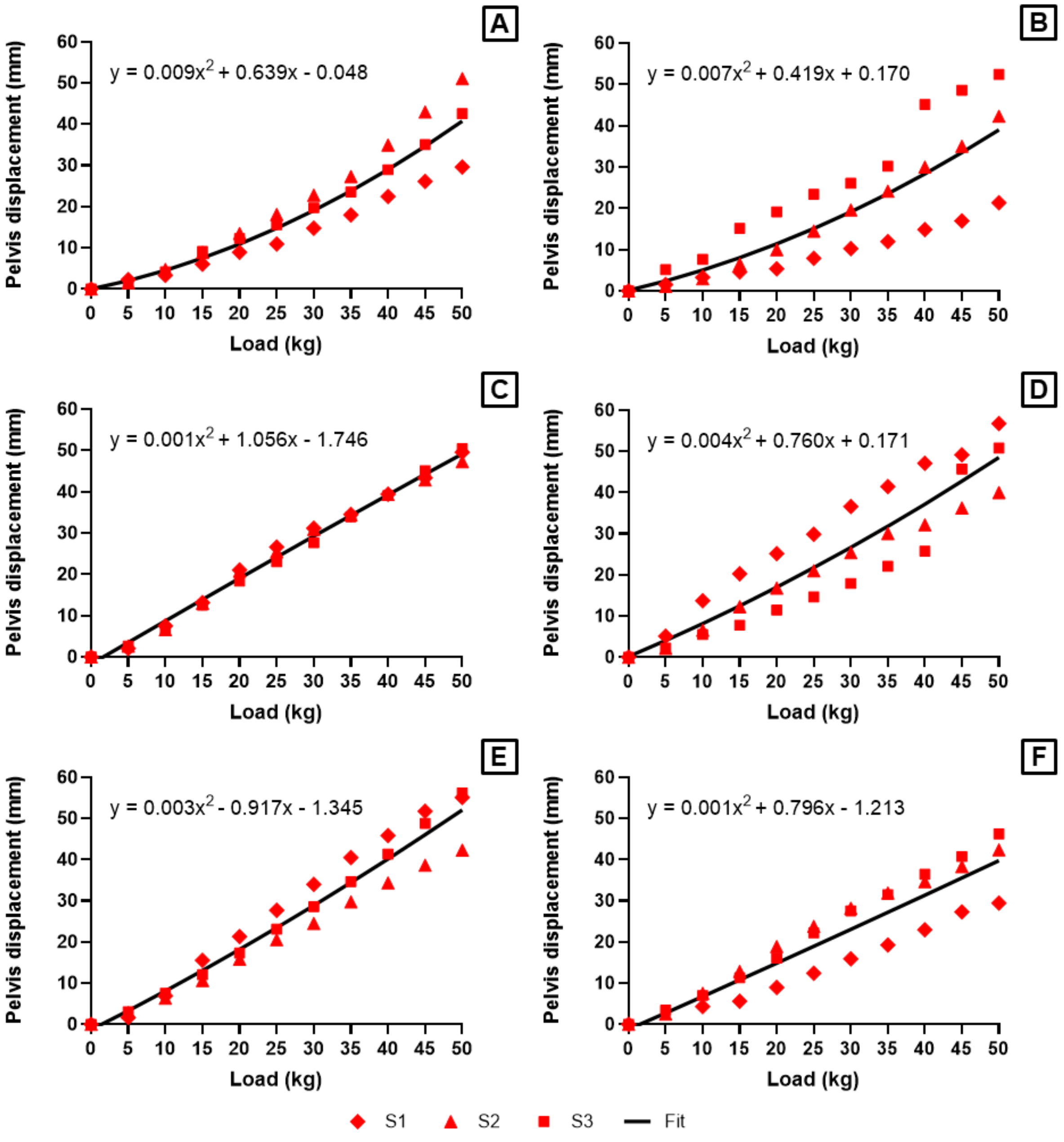

Figure S1: Quadratic regression model of load-displacement points at three inclinations of the operating bed: experimental data with fitted curve and equation. (A): PinkPad at 0°. (B): CarePad at 0°. (C): PinkPad at 5°. (D): CarePad at 5°. (E): PinkPad at 10°. (F): CarePad at 10°.

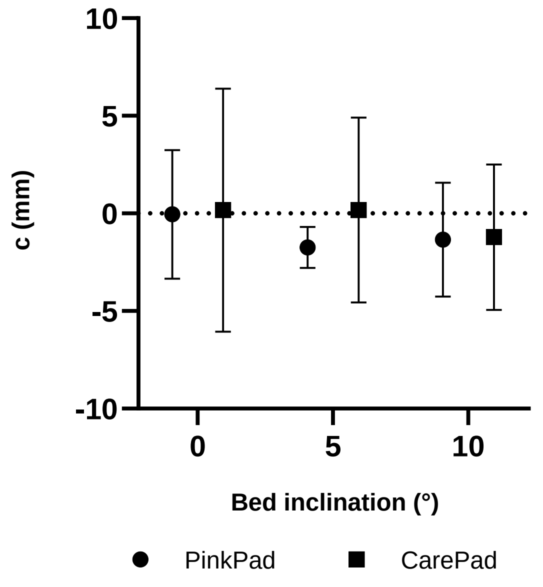

Figure S2: Intercept of the quadratic regression model of load – displacement points at three inclinations of the operating bed. Estimated value and 95% CI of the parameter for the three subjects are represented.

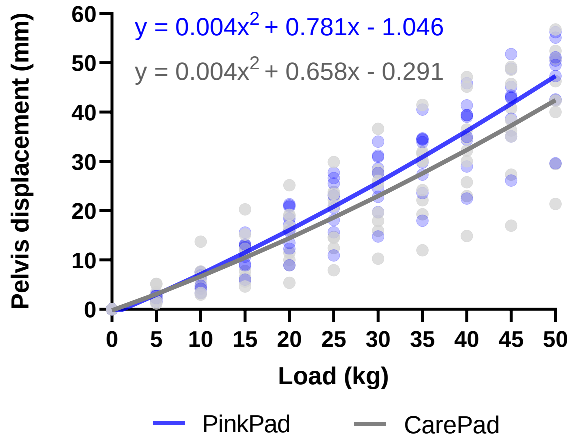

Figure S3: Quadratic regression model of load-displacement points considering all inclinations of the operating bed: experimental data with fitted curve and equation.

Table S1: Traction loads for achieving hip distraction from literature.

Case study	Traction load (kg)
Eriksson et al., 1986 [1]	31 – 51
Arvidsson, 2020 [2]	> 41
Grøntvedt & Engebretsen, 1995 [3]	20 – 41
Elsaidi et al., 2004 [4]	> 20
Smart et al., 2007 [5]	23 – 28
Sabetta & Ferraù, 2010 [6]	25 – 40

Telleria et al., 2012 [7]	40 – 57
Martin et al., 2012 [8]	26
Ellenrieder et al., 2017 [9]	49
Röling et al., 2020 [10]	48
Mortensen et al., 2023 [11]	45

Table S2: Traction loads for achieving femoral shaft fracture reduction from literature.

Case study	Traction load (kg)
Winquist et al., 1984 [12]	16 – 20
Kruger et al., 1990 [13]	35
Brumback et al., 1992 [14]	35
Gösling et al., 2006 [15]	< 40
Maeda et al., 2008 [16]	32

References

[1] E. Eriksson, I. Arvidsson, H. Arvidsson, "Diagnostic and Operative Arthroscopy of the Hip," *Orthopedics*, vol. 9, no. 2, pp. 169–176, 1986, doi: 10.3928/0147-7447-19860201-07.

[2] I. Arvidsson, "The hip joint: forces needed for distraction and appearance of the vacuum phenomenon," *J. Rehabil. Med.*, vol. 22, no. 3, pp. 157–161, 2020, doi: 10.2340/165019779022157161.

[3] T. Grøntvedt, L. Engebretsen, "Arthroscopy of the hip," *Scand. J. Med. Sci. Sports*, vol. 5, no. 1, pp. 7–9, 1995, doi: 10.1111/j.1600-0838.1995.tb00003.x.

[4] G. A. Elsaidi, D. S. Ruch, W. D. Schaefer, K. Kuzma, B. P. Smith, "Complications associated with traction on the hip during arthroscopy," *J. Bone Joint Surg. Br.*, vol. 86-B, no. 6, pp. 793–796, 2004, doi: 10.1302/0301-620x.86b6.14426.

[5] L. R. Smart, M. Oetgen, B. Noonan, M. Medvecky, "Beginning Hip Arthroscopy: Indications, Positioning, Portals, Basic Techniques, and Complications," *Arthrosc. J. Arthrosc. & Relat. Surg.*, vol. 23, no. 12, pp. 1348–1353, 2007, doi: 10.1016/j.arthro.2007.06.020.

[6] E. Sabetta, C. Ferraù, "L'artroscopia dell'anca," *LO SCALPELLO-OTODI Educ.*, vol. 24, no. 3, pp. 190–197, 2010, doi: 10.1007/s11639-010-0083-1.

[7] J. J. M. Telleria, M. R. Safran, J. N. Gardi, A. H. S. Harris, J. M. Glick, "Risk of sciatic nerve traction injury during hip arthroscopy - Is it the amount or duration? An intraoperative nerve monitoring study," *J. Bone Jt. Surg.*, vol. 94, no. 22, pp. 2025–2032, 2012, doi: 10.2106/JBJS.K.01597.

[8] H. D. Martin, I. J. Palmer, K. Champlin, B. Kaiser, B. Kelly, M. Leunig, "Physiological Changes as a Result of Hip Arthroscopy Performed With Traction," *Arthrosc. J. Arthrosc. & Relat. Surg.*, vol. 28, no. 10, pp. 1365–1372, 2012, doi: 10.1016/j.arthro.2012.04.139.

[9] M. Ellenrieder, T. Tischer, R. Bader, P. C. Kreuz, W. Mittelmeier, "Patient-specific factors influencing the traction forces in hip arthroscopy," *Arch. Orthop. Trauma Surg.*, vol. 137, no. 1, pp. 81–87, 2017, doi: 10.1007/s00402-016-2572-z.

[10] M. A. Röling, N. M. C. Mathijssen, I. Blom, T. Lagrand, D. Minderman, R. M. Bloem, "Traction force for peroperative hip dislocation in hip arthroscopy," *HIP Int.*, vol. 30, no. 3, pp. 333–338, 2020, doi: 10.1177/1120700019841579.

[11] A. J. Mortensen, A. K. Metz, J. Featherall, D. C. O'Neill, R. M. Rosenthal, S. K. Aoki, "Hip Joint Venting Decreases the Traction Force Required to Access the Central Compartment During Hip Arthroscopy," *Arthrosc. Sport. Med. Rehabil.*, vol. 5, no. 3, pp. e589–e596, 2023, doi: 10.1016/j.asmr.2023.02.011.

[12] R. A. Winquist, S. T. Hansen, D. K. Clawson, "Closed intramedullary nailing of femoral fractures. A report of five hundred and twenty cases.," *J. Bone & Jt. Surg.*, vol. 66, no. 4, pp. 529–539, 1984, doi: 10.2106/00004623-198466040-00006.

[13] D. M. Kruger, D. C. Kayner, F. M. Hankin, M. H. Falahee, H. Kaufer, L. S. Matthews, S. A. Goldstein, "Traction Force Profiles Associated with the Use of a Fracture Table: A Preliminary Report," *J. Orthop. Trauma*, vol. 4, no. 3, pp. 283–286, 1990, doi: 10.1097/00005131-199004030-00008.

[14] R. J. Brumback, T. S. Ellison, H. Molligan, D. J. Molligan, S. Mahaffey, C. Schmidhauser, "Pudendal nerve palsy complicating intramedullary nailing of the femur.," *J. Bone Jt. Surg.*, vol. 74, no. 10, pp. 1450–1455, 1992, doi: 10.2106/00004623-199274100-00003.

[15] T. Gösling, R. Westphal, J. Faülstich, K. Sommer, F. Wahl, C. Krettek, T. Hufner, "Forces and torques during fracture reduction: Intraoperative measurements in the femur," *J. Orthop. Res.*, vol. 24, no. 3, pp. 333–338, 2006, doi: 10.1002/jor.20045.

[16] Y. Maeda, N. Sugano, M. Saito, K. Yonenobu, I. Sakuma, Y. Nakajima, S. Warisawa, M. Mitsuishi, "Robot-assisted femoral fracture reduction: Preliminary study in patients and healthy volunteers," *Comput. Aided Surg.*, vol. 13, no. 3, pp. 148–156, 2008, doi: 10.3109/10929080802031038.
